# Midwives’ perceived role in up referral of high-risk pregnancies in primary healthcare settings, eThekwini district, South Africa

**DOI:** 10.4102/hsag.v26i0.1546

**Published:** 2021-02-25

**Authors:** Siyabonga W. Ximba, Olivia B. Baloyi, Mary Ann Jarvis

**Affiliations:** 1 Department of Nursing, College of Health Sciences, University of Kwazulu-Natal, Durban, South Africa

**Keywords:** high-risk pregnancy midwives, primary healthcare, up referral, eThekwini, South Africa

## Abstract

**Background:**

South African maternal and neonatal mortality rates remain unnecessarily high, which are considered avoidable through timeous identification, treatment and referral. An efficient referral process of high-risk pregnant women is dependent on the midwives’ ability to respond with relevance to the maternal and neonatal healthcare needs. The attainment of improved maternal and neonatal outcomes commences at the primary healthcare level, with the midwife, recognised as the first person responsible and accountable for pregnant women’s healthcare.

**Aim:**

To explore midwives’ perceptions of their role in the referral of high-risk pregnant women from primary health care clinics to expert-centred sites.

**Methods:**

A qualitative, exploratory, descriptive in nature approach, underpinned by a social constructivism paradigm, guided the methodology. Purposive sampling was used to select both the primary health care clinics in the feeder zone and the registered midwives working in these clinics. Data were collected through four focus group discussions and analysed using content analysis. The principles of trustworthiness were observed.

**Setting:**

Department of Health primary health care clinics in the eThekwini district, South Africa.

**Results:**

The midwives understood their role in the up referral of high-risk pregnant women but experienced many interruptions in its execution. Four categories emerged from the data, namely, enhanced by team support in the clinics, restrictions in transfer to expert-centred sites, impeding social determinants and midwifery competence facilitates referral process.

**Conclusion:**

Global initiatives cannot guarantee maternal and neonatal health because of the challenges experienced by the midwives in the execution of their roles as they interface with the healthcare team.

## Introduction

Globally 830 maternal deaths occur daily, 99% in lower-middle-income countries (LMICs) (WHO 2019) of which 87% occur in South Asian and sub-Saharan African regions (Biswas et al. 2018), inclusive of South Africa with a maternal mortality rate of 134 per 100 000 live births (Moodley, Fawcus & Pattinson 2018; WHO 2019). Maternal mortality does not occur in isolation, as it is coupled with reports of 3.7 million neonatal deaths globally, 75% occurring in the first week of life (UNICEF 2019). A key global initiative to prevent maternal and neonatal deaths ensuring prompt interventions in the event of complications has been the introduction of skilled birth attendants at the point of delivery (LSTM 2018).

Despite the global goals, South African maternal and neonatal mortality rates remain unnecessarily high (National Department of Health [NDoH] 2018), which are considered avoidable through timeous identification, treatment and referral (Biswas et al. 2018; Pacagnella et al. 2014). In an attempt to provide for safer healthcare delivery, the South African government adopted the primary healthcare (PHC) approach, followed by a tiered classification of healthcare with clear protocols for the management of complications, and specific referral pathways (NDoH 2016). The referral pathway in South African maternity services usually follows the route from the PHC setting (feeder area) to Level-1 hospitals to Level-2 hospitals (expert-centre sites) depending on the level of severity of the presenting condition and the necessity to access an obstetrician and/or paediatrician (NDoH 2016). A system of clear referral pathways should always be in place, so that pregnant women who require high levels of care are managed and treated by appropriate specialist teams when complications are identified (NICE 2019).

An efficient referral process is dependent on the skilled midwives’ ability to respond with relevance to the maternal healthcare needs. The South African NDoH has responded to this requirement through providing workshops to midwives on the guidelines for maternity care and the Essential Steps in the Management of Obstetric Emergencies (ESMOE) (Frank, Lombaard & Pattinson 2009). The aforementioned changes were goaled at improving pregnant women’s access to antenatal services, appropriate utilisation of different levels of care and improved maternal health (NDoH 2016).

The attainment of improved maternal outcomes commences at the PHC level, with the midwife, recognised as the first person responsible and accountable for pregnant women’s healthcare (Mmusi-Phetoe 2016). The midwives in the PHC clinics are accountable for the promotion of normal birth, the detection and prevention of complications for mother and baby, the implementation of emergency measures and referral where necessary (Beek, McFadden & Dawson 2019; Pacagnella et al. 2014). At various stages of pregnancy, labour or the puerperium, the health of pregnant women or the foetus is threatened, classifying them as at risk (NIH 2010). Examples of complications that might present in pregnancy and labour and are deemed high risk and requiring referral include hypertension, diabetes, anaemia, previous caesarean section (NICE 2019), haemorrhage and cephalopelvic disproportion (Haddrill et al. 2014).

The South African Nursing Council (SANC) legislative documents outline that the midwife shall subject to the provisions of sub-regulation R2598 refer the patient to a higher level of care in the event of abnormalities or complications occurring during pregnancy and labour (SANC 1991). The midwife plays a significant role in timeous referrals; late referral from PHC settings have contributed to the increase in maternal and neonatal mortality ratios at Levels 1 and 2 hospitals, as expert-centred sites employing obstetricians (NDoH 2018). Little is known about midwives’ perceptions of their role as central in the referral process from the PHC clinic to the next level of care, given that an interruption in the referral process could contribute to an increase in maternal and neonatal mortality ratios.

## Aim

To explore midwives’ perceptions of their role in the referral of high-risk pregnant women from PHC clinics to expert-centred sites.

## Research design and method

### Study design and approach

A qualitative, exploratory, descriptive in nature approach, underpinned by a social constructivism paradigm, guided the methodology. The belief was that this approach would provide an in-depth understanding of the midwives’ understanding of their roles in the referral process.

### Study setting

Eight PHC clinics were selected as feeder sites (points for up referral to the next level) to the Level 1 hospital, because of the possible link between the rising neonatal mortality rates and the late up referral. The maternal/neonatal record reviews from the Level-1 hospital highlighted that in the last half of 2018, 82% of women were referred from feeder sites. All the referred women presented with complications such as pre-term labour, pre-eclampsia, prolonged labour and abruptio placentae. Of the 41 stillbirths delivered at the hospital during that period, 70.7% was referrals from the feeder sites and 29.3% was attending at the hospital. In addition within the same period, of the 18 early neonatal deaths at the hospital, 72.2% was referrals from the feeder sites and only 27.8% was attending at the hospital. The possible link between the rising neonatal mortality rates and the late referral was evidenced by maternal/neonatal record reviews which highlighted that some woman were kept for longer at the PHC settings, where the second stage of labour was already prolonged.

These eight PHC clinics are government institutions located within the western semi-urban and rural areas of eThekwini district municipality. The furthest of the feeder clinics is 32.4 km from the Level 1 hospital. Three of the feeder PHC settings offer 24-hr maternal services, including deliveries, whilst the other five only offer day maternal services. The services offered at PHC settings are not limited to maternal healthcare but are comprehensive in nature, and the people living in the feeder areas are reliant on the provided healthcare services.

The most frequent occupation of the pregnant women is domestic employment with minimal daily or weekly earnings, because of low levels of education and skills, which negatively affects the standards of living. The occupation of most men in this area is taxi conductors or roadside labourers with small earnings, affecting their access to basic needs, or a decent shelter.

### Study participants and sampling

Purposive sampling was used to select the eight PHC clinics in the feeder zone to the Level 1 hospital, as nearly three quarters of admissions were up referrals, with a rising neonatal mortality rate. Further, purposive sampling was used to select the registered midwives working in these clinics as they are the front-line healthcare providers for maternal healthcare services, at a PHC level and their perceptions need to be made visible. Both categories of midwives (advanced midwives [AM] and basic midwives [BM]) were represented in the PHC clinics, with every PHC clinic having at least one advanced midwife who had a post-basic qualification, in addition to the basic midwives with an undergraduate midwifery qualification. On average each PHC clinic had four registered midwives per shift, one of whom was an advanced midwife. All registered midwives in maternity at the select PHC clinics, who were willing to participate and on duty on the day of data collection were invited to participate in the study.

### Data Collection

Data were collected in English as the reporting language, between December 2019 and March 2020, in the respective clinics, through focus group discussions (FGDs), which with participants’ written consent were audio recorded. Each FGD consisted of between four and six registered midwives and lasted between 45 min and 90 min. Data saturation was achieved after the third FGD, and the fourth FGD allowed for confirmation of data and to ensure no new categories emerged. An open-ended question based on the interview guide was asked:

‘As a midwife working in the PHC clinic, rendering maternity healthcare services, please share with us what is your perceived role in the referral process of high-risk pregnant women to the higher level of care.’

#### Trustworthiness

The principles of trustworthiness, credibility, dependability, confirmability and transferability (Lincoln & Guba 1985) guided the data collection and analysis. To ensure credibility of the data, two researchers together collected the data, one an expert midwife and the other an expert psychiatric nurse with interview skills, followed by member checking. The clearly articulated research proposal determined dependability of the study. The researchers analysed the data from the four focus groups through independent coding, triangulation of the data followed by discussions and consensus to arrive at the categories and sub-categories, thereby ensuring confirmability. The description of the context of the PHC clinics and the tiered health system, together with the data collection and analysis procedures, evidenced transferability.

#### Data analysis

The data from the four FGDs were analysed using the inductive approach of the three-phase content analysis by Elo and Kyngas (2008). The initial preparation phase (Elo & Kyngas 2008) involved deciding to use FGDs as the unit of analysis. Organising followed as the second phase (Elo & Kyngas 2008) and involved transcription and coding. The voice recordings were listened to separately and several times by the researchers, which facilitated immersion in the data. This was followed by coding until the emergence of categories and sub-categories representing the initial concepts and thoughts. The initial analysis was put onto the coding sheet and discussed by the researchers until consensus was reached. All data within a sub-category were examined to ensure a fit between the data and the sub-category. Sub-categories were grouped into emergent categories through agreement. The researchers reviewed the analysis and compared the codes to the transcripts. The third phase was reporting (Elo & Kyngas 2008), which involved presentation of the results.

### Ethical considerations

The University of KwaZulu Natal, Humanities and Social Sciences Research Ethics Committee granted ethical clearance (HSSREC/00000388/2019). Permission to conduct the study was further sought from all relevant authorities, that is, KwaZulu-Natal Department of Health and Managers of respective PHC clinics. Prior to the fieldwork the participants received an information sheet explaining the study. Written consent was obtained from all the participants for both inclusion in the study and audio recording. Participants received assurance of the voluntary nature of the study that they could withdraw from the study at any given time without any repercussions and that anonymity and confidentiality would be maintained at all times.

## Results

### Profile of the participants

Seventeen registered midwives participated in the study across four PHC centres in different locations in a select district of eThekwini. The fourth group was for the confirmation of data. The years of experience as a registered midwife ranged from 2 to 34 years with the average being nine years.

### Study findings

The midwives understood their role in the referral process from the PHC clinics but experienced many interruptions in its execution. Four categories emerged from the data, with the midwives mainly perceiving their role in the referral process of transferring high-risk pregnant women to be impeded as opposed to enhanced (Table 1). They only perceived one enhancing factor, whilst both the processes linked to the healthcare structures and the patient created obstacles, preventing the execution of a seamless transfer (Table 1). However, evidence highlighted their competence and ability to action their maternal healthcare knowledge, emphasising their understanding of their role (Table 1).

**TABLE 1:** Categories and sub-categories.

Categories	Sub-categories
Category 1: Enhanced by team support in the clinic	Sub-category 1A: Collegial mentorship facilitating timeous referral
Category 2: Restrictions in the transfer to expert-centred sites	Sub-category 2A: Interruption in the communication loop
	Sub-category 2B: Interrogation of professional decision-making
	Sub-category 2C: Referral transportation delays
Category 3: Impeding social determinants	Sub-category 3A: Financial limitations
	Sub-category 3B: Lower maternal health literacy
Category 4: Midwifery competence facilitates referral process	Sub-category 4A: Independently triaging maternal health risk
	Sub-category 4B: Actioning the maternity care guidelines

### Category 1: Enhanced by team support in the clinic

All participants identified support from their peers as beneficial to the referral process. The peer support increased the participants’ ability to cope with the level of skill required in the management of the high-risk pregnant women, thereby facilitating the referral and ease in exercising their role.

#### Sub-category 1A: Collegial mentorship facilitating timeous referral

Mentorship was identified by the participants as a major role player in the identification and timeous management of the high-risk pregnant women and in the referral process. It emerged from the data sources that whenever a junior midwife realised the confines of her role and the need for advanced help, an advanced midwife would intervene. If the advanced midwife could not manage further, they received guidance from the doctor at the next level of care, the referral hospital. This cascade of collegial mentorship is illustrated in the following extracts:

‘[*A*]s an advanced midwife I also have a role of mentoring our junior midwives, as they manage their own patients where they seem unsure, they call me where I assist in assessing the patient and advise accordingly …’ (FG3 PC AM)

‘[*A*]s someone who is still junior I find the help or rather the consultation with advanced midwives very helpful in ensuring that patients with complications or those who cannot be managed in our PHC clinic are referred on time without further delays and complications.’(FG3 PD BM)

‘[*A*]dvanced midwives we don’t know everything, sometimes we also don’t know what is wrong with the patient or what to do, then I also consult doctor at the another hospital, which is usually our referring hospital for further guidance.’ (FG4 PB AM)

### Category 2: Restrictions in the transfer to expert-centred sites

The participants described encountering various challenges in their referral process of high-risk pregnant women to expert-centred sites, as they attempted to perform their role.

#### Sub-category 2A: Interruption in the communication loop

Participants indicated that high-risk patients presented at their PHC facility for delivery, even though earlier in their pregnancy they had been referred to the expert-centred site. They further alluded that this put both the mother and the baby at an even higher risk. These were some of the comments from the participants:

‘[*C*]ommunication breakdown is the big issue which interferes with the referral processes.’ (FG2 PC AM)

‘[*W*]e communicate with our patient in isiZulu which is the medium language of communication but still they will come back here with their conditions worsened (FG1 PC AM) which their life and that of their unborn child at risk.’ (FG1 PE BM)

It also emerged from the participants that the clinics’ phone and receiving hospital’s phones were often faulty, necessitating the use of their own private mobile phones, as shown in the extracts below.

‘[*S*]ometimes their phones are faulty. They’ve got only cell phones, we have to use our cell phones … most of the time in Level-1 their labour ward phone is not working which makes it difficult to communicate.’ (FG1 PC AM)

‘[*S*]ometimes you can spend three hours trying to get hold of the referral hospital (FG3 PB BM) … what happens then if you have severe fetal distress the baby will die whilst busy trying to phone.’ (FG3 PA AM)

The participants acknowledged the boundaries of their roles and the need to up refer. They further highlighted the hurdles they experienced in the referral process, when trying to communicate with the doctors. They were not updated on changes in the doctors’ roster, consequently calling an off-duty doctor and delaying the referral process:

‘[*G*]etting hold of doctors is also a nightmare … they provide us with rosters for doctors but we never informed when the roster changes you end up spending your last airtime to call someone who is off or “[*N*]ot on call.”’ (FG1 PD AM)

‘[*T*]his is not only frustrating but also delays our referral time and the patient is also deteriorating.’ (FG4 PB AM)

All participants further stated that it was a challenge to communicate with the emergency transport department; it could take up to an hour on the phone waiting to be assisted.

‘[C]ommunication with emergency transport department is a huge challenge (FG4 PB AM) … we hold for an hour trying to get through (FG3 PB BM) … they never respond on time.’ (FG 2 PD BM)

The participants highlighted the frustration, when timeously trying to connect with the emergency transport services by phone, further complicated by them arriving unprepared. They would come without emergency equipment such as an incubator, resuscitation equipment or advanced personnel necessary to transport the patient to specialised care, which further interfered with the implementation of their role and the referral process, as indicated in the statements below:

‘[*M*]mm you get through to them after an hour of holding you explain to them that maybe you have an asphyxiated neonate who needs to be transferred in an incubator in the presence of an advanced paramedic. No they will come unprepared pulling a striker for an adult (FG2 PC AM)…no incubator no advanced paramedic … then we back to square one the patient cannot be transferred.’ (FG4 PB AM)

#### Sub-category 2B: Interrogation of professional decision-making

Most participants expressed strong levels of frustration with how some doctors from the referral hospitals interrogated their decision, even asking for photographic evidence, when they sought to up refer a patient, despite their verbatim referencing of the maternity care guidelines. At times when their knowledge was questioned and the doctor did not want to accept the patient, it resulted in them touting their qualifications to the referring doctor, or reiterating the correctness of their referral by placing a copy of the guidelines in the patient’s file to present at the hospital. The subsequent extracts provide evidence of the midwives’ confidence of their role in the face of the interrogation:

‘[*T*]he interrogation and undermining we suffer at the hands of the doctors where we are referring our patients is awful … it’s bad very bad (FG1 PC AM) … the cross questioning is terrible (FG4 P4 AM) … Are you sure that is foetal distress … take a picture of the CTG strip and send it.’ (FG1 PA BM)

‘[*I*]t doesn’t matter whether you are an Advanced Midwife or junior midwife the treatment is the same the interrogation is the same … this one time I felt like this is enough I just photocopied the maternity guidelines, pin on the patient’s chart and transferred.’ (FG1 PC AM)

#### Sub-category 2C: Referral transportation delays

All the participants strongly agreed not only on the interruption of the communication loop with the emergency transport but also on major delays experienced with their arrival. The changing of shifts in the emergency medical services delayed their arrival and resulted in further complication in the patient, whilst the oncoming ambulance staff started by washing the ambulance, despite the emergency. They indicated that the change in shifts and the delay in ambulances had contributed largely to complicated maternity cases not reaching the hospital timeously. These challenges are evident from the following quotes:

‘[*T*]heir turnaround time is very long for instance I’ve had a case once where EMRS was phoned as early as 8 o’clock am and they only came maybe at 14:00 pm by the time they reached here the patient condition had deteriorated.’ (FG1 PA BM)

‘[*I*]f you requested an ambulance closer to change over time forget it you will even go off still waiting for it to arrive (FG2 PC AM) … the coming in shift start by washing the ambulance … which adds to the waiting time you literally looking at this bleeding woman or dropping foetal heart rate and there is nothing more to do.’ (FG3 PB BM)

### Category 3: Impeding social determinants

The midwives would have executed their role, but the social determinants specific to the patients accessing the healthcare setting impacted on their access to the referral site.

#### Sub-category 3A: Financial limitations

It emerged from the data sources that despite the registered midwives’ referral of the high-risk patients to the next level of care and providing budget counselling about transport monies, financial constraints limited the patients accessing the expert-centred sites; consequently, the patients presented at their PHC sites for delivery. The following extracts support this statement:

‘[*O*]ur patients come from not so good social background like XX and YY which affects their health seeking behaviour (FG1 PB BM) … usually what happens is when they have a high-risk condition which is not urgent like being overweight we always ask them to go the next level of care the following day and most of the time they don’t go because they have no financial means to get there (FG2 PC AM) … they end up coming back to us for delivery which is very problematic we have to book transport for them because most of the time their reason they say they don’t have money.’ (FG4 PD BM)

#### Sub-category 3B: Lower maternal health literacy

The midwives understood their role for referral, but the problem arose from the pregnant women’s low maternal health literacy. It emerged from the data sources that low maternal health literacy, involving a lack of understanding of the serious risk factors such as teenage pregnancy, previous caesarean section and grand multipara led to patients’ poor understanding of the referral system. The literacy levels were further influenced by both family and community members. The patients failed to understand the risk of their pregnancy and based their perceptions of the facility’s adequacy on community members’ successful deliveries. The extracts below confirm how lower maternal literacy and community influence have a negative impact on the patients’ health-seeking behaviour and the midwives’ ability to fulfil their role:

‘[*T*]hey do not have only financial problems but they are also uneducated, majority do not have basic secondary education (FG1 PD AM) … Because of their low literacy they are unable to understand the seriousness of their conditions, they have no insight of their conditions … let alone that they cannot be delivered in PHC they come back even when they were referred.’ (FG1 PE BM)

‘[… *S*]ome patients would come even if you have explained everything that they should go to the hospital, but they would come and say “my mother said I must come here” and you see the mother outside (FG4 P3 BM) … they do not understand the different levels of care to them is like nurses are nurses everywhere so why leave a nurse next to my house and go to XXX hospital (FG2 PC AM) … they don’t understand that we don’t have the necessary resources to assist them (FG2 PB BM) … like no theater at PHC therefore no caesarian sections can be performed here.’ (FG1 PB BM)

### Category 4: Midwifery competence facilitates referral process

All participants identified that their ability to independently triage and conduct practice as per the guidelines were skills that contributed positively to the referral process.

#### Sub-category 4A: Independently triaging maternal health risk

The participants identified that as part of the referral process, at the first antenatal visit, the identification of high risk required a heightened level of skill, knowledge and vigilance, as they triaged patients according to the severity of the presenting pregnancy condition into three categories, low-risk, high-risk and then very high-risk. The triaging facilitated referral to different levels of care. Some of the participants stated:

‘[*W*]e are the first point of entry for pregnant women …. they report to once they discover they are pregnant … it is at this first antenatal visit where we triage them … screen and grade them as either low-risk, high-risk or very high risk. (FG3 PA AM) … to further determine who stays with us at PHC or who needs to be referred to the next level of care.’ (FG3 PD BM)

‘[… *I*] mean the triaging it doesn’t come easy it draws in itself some skills, knowledge and vigilance from us as midwives.’ (FG3 PA AM)

The participants went on to highlight that the referral to the next level of care was determined by the severity of the presenting condition, where they further classified the patients as elective or emergency referrals. At times, it meant referral directly to a Level 2 hospital. The participants stated:

‘[… *Y*]ou triage the case … assess whether it’s an elective referral or emergency referral. If a patient comes now presenting with imminent eclampsia we won’t direct that patient to … to district level because they don’t manage those cases, you direct straight to secondary level because if that imminent eclampsia had occurred at the district hospital they themselves would’ve sent straight secondary level …’ (FG 2 PB BM)

#### Sub-category 4B: Actioning the maternity care guidelines

All participants were specific in describing their actions with direct reference to the national maternity care guidelines whenever they were referring the patient to the next level of care. The guidelines stipulate what needs to be implemented at the PHC level and what needs to be carried out once they have exhausted all those interventions. Participants were knowledgeable on the different levels of care and referral pathways as patients were up referred, according to their graded level of risk. Participants elaborated:

‘[… *M*]aternal Care Guidelines guides our referral process it states exactly who is to be treated where PHC, Level 1 or Level 2 … according to the severity of the condition (FG2 PC AM) … it also guides what to do while waiting for referral.’ (FG1 PA BM)

## Discussion

The study’s aim was to explore and describe the midwives’ perceived role in the transfer of high-risk pregnant women from the PHC setting to the next level of expert care. It highlighted the midwives’ feelings of competence towards their role in the transfer process; however, their ability to execute their roles encountered interruptions from various obstacles in their engagement with physical, healthcare user and organisational characteristics. The interruptions in the transfer process resulted in negative outcomes to both maternal and neonate health as well as to the morale in the work environment of the PHC clinic (Stievano et al. 2018). Donabedian (2005) in his model of evaluating quality care depicted that structure measures have an effect on process measures, which in turn affect the outcome measures.

This study evidenced that the midwives, who formed part of the structure measures in Donabedian’s model (2005), were well prepared to execute their role in meeting the needs of high-risk pregnant women entering the PHC clinics, from a healthcare context of a quadruple burden of disease. This finding is in contrast to the studies in LMICs which show that 66.7% of avoidable factors for maternal and neonatal deaths is linked to healthcare workers, where midwives at PHC level lacked the necessary knowledge for referral (Merali et al. 2014). In South Africa, a tiered healthcare system (NDoH 2018), legislative documents (NDoH 2016; SANC 1991), upskilling of midwives and ESMOE training (NDoH 2017) are amongst the measures that have been put in place to support midwives in practice and simultaneously address the unacceptably high maternal and neonate mortality rates (Moodley et al. 2018; NDoH 2018; WHO 2019).

However, in this study, despite the preparedness of the midwives and the confidence they exuded towards knowing the Maternity Care Guidelines, this was not the case for other organisational structures. The midwives had to contend with interruptions in the communication loop, such as non-functional telephones, delays in answering of phone calls and rosters that were not updated. This problem is not unique to South Africa. Austin et al. (2015) examined communication and feedback within the maternity referral system in Addis Ababa and identified the lack of a dedicated communication system, faulty telephones and radio systems restricted the midwives in executing the referral protocol. The structure obstacles to transferring the pregnant women to the expert site were not just confined to the organisational structures, as they included the healthcare users’ lower maternal health literacy and financial restraints. The patient’s cooperation is a key factor in accomplishing the desired healthcare outcome (Mosadeghrad 2014), which can be thwarted by the patient’s lack of understanding of the reason for referral (Harahap, Handayani & Hidayanto 2019). Other local and African studies have shown that financial constraints, such as the inability to obtain transportation monies, have been the major contributors towards the non-compliance in the referral process (Harahap et al. 2019; Pembe et al. 2010; Tsawe & Susuman 2014).

The process component of Donabedian’s (2005) model of quality care focuses on the care delivered to the healthcare user and holds relevance in this study examining the midwives’ perceptions of their role in the transfer of the high-risk pregnant women to a higher level of care. Although the midwifery team were small in numbers, with varied years of expertise, they always included an advanced midwife, and worked as a team, providing support and mentorship to each other, centred on the common goal of maternal and neonate health, inclusive of timeous up referral. Various authors (Govender, Naidoo & Taylor 2019; Siassakos et al. 2013), in agreement with this study’s findings, reported that working together as healthcare workers improves communication, facilitates the prompt identification of high-risk patients and timeous referral to the appropriate care.

The assumption is that the collegial relationships contributed to the midwives’ understanding of their role in identifying the characteristics of high-risk pregnancies. The midwives were confident in their ability to triage according to the maternity care guidelines, in an attempt to avert an emergency. Midwives’ ability to implement the maternity care guidelines is vital to the successful delivery of maternal healthcare, in contrast to the studies that have revealed midwives ‘not knowing what they did not know’ (Ojuri-King, Jarvis & Baloyi 2018).

In contrast to this study, the midwives’ conviction of ‘knowing what they knew’ (Lynch 2017) about the maternity care guidelines drove them to persevere in reaching out to the structures involved in the referral process. Their perseverance was often met head-on with an interruption of the communication loop, intra-professional disrespect, which was evident in the disregard of the midwives’ requests to the emergency medical services and interrogation of their professional decision-making, further complicated by the late arrival of the emergency medical services. Hussein et al. (2012) highlighted that good communication, availability of emergency transport, geographical access to care and the availability of health services as factors that affect a successful referral system; whilst conflict between midwife and doctor, in particular the resistant attitude by doctors towards inter-collaboration can negatively influence communication (Downe, Finlayson & Fleming 2010). The barriers the midwives experienced in exercising their role were not unique to the South African healthcare context. Reiger and Lane (2009) identified in an Australian study that the participant midwives felt marginalised, disrespected and that their clinical judgement when referring pregnant women was doubted by the doctors. In contrast, it is noteworthy that collaboration between midwives and doctors has a positive outcome on maternal health (Matthys, Remmen & Van Bogaert 2017).

The structures and the processes in Donabedian’s tripartite model of quality care point towards the outcome on the health status of the patient. In this study, the outcome of the disregard by other healthcare personnel, and also patient’s disregard of the midwives’ professional decisions, resulted in complications and deterioration of maternal and neonate health as transfers were delayed. The National Committee for Confidential Enquiry into Maternal Deaths (NCCEMD) of South Africa (2017) identified ambulance issues as one of the factors related to the deaths, further stating that these deaths are preventable by timely emergency transportation. International studies further support the findings of the NCCEMD and the contribution of ambulance delays to poor maternal health outcomes (Bomela 2020; Pacagnella et al. 2014).

## Limitations

The study setting was limited to one geographical area of eThekwini and, therefore, findings generalisable with due consideration.

## Recommendations

The use of designated obstetric emergency transport as recommended by the NCCEMD (2017) should be carefully monitored.

The creation of an interdisciplinary platform where community representatives, emergency medical services, midwives and obstetricians meet to gain an understanding of the midwives’ role in the transfer of high-risk pregnant women to expert-centred sites.

There is a need for PHC clinics to develop programmes for community involvement and engagements. This will sensitise the community on the services offered at the clinic, thereby preventing high non-compliance with the referral.

Additional research is required on the impact of the inappropriate referrals on the quality of maternal care services.

## Conclusion

This study highlighted that the research sites met the WHO (2019) recommendation of skilled birth attendants at the point of delivery. The midwives were confident in their role of the transfer of high-risk pregnant women to the expert sites as outlined in the maternity care guidelines (NDoH 2016). However, such global initiatives (WHO 2019) cannot guarantee maternal and neonatal health because of the challenges experienced by the midwives in the execution of their roles as they interfaced with other parties. Within a context of high maternal and neonate mortality rates, in order to achieve Sustainable Developmental Goals ([SDG] 3.1 and 3.2), these challenges need to be prioritised on local and international healthcare agendas to ensure a seamless, timeous transfer of high-risk pregnant women.
